# Forty Years an Advertising Agent

**Published:** 1906-01

**Authors:** 


FORTY YEARS AN ADVERTISING AGENT. By George P. Rowell; 550 pages octavo; illustrated. Printers' Ink Publishing Co., New York. Price, $2.00.

It is with pleasure we make public a preliminary announcement concerning the above book, which will undoubtedly prove of value as a book of reference for many years. It treats of a subject no one else has made any careful attempt to deal with.

---

### EVERY LITTLE BIT HELPS.

"One of the spiciest little journals that comes to the secretary is the *American Medical Journal.* It contains many very readable articles, and its last issue seems to be chiefly directed against the various transactions of the American Medical Association, especially against the *Journal.* We rather think that there is a great deal of truth in what it has to say and advise all who wish to know both sides of the question to read it. It is published by D. A. O'Gorman, of New York."—From the *Journal of the South Carolina Medical Association* (published under the direction of the Publication Committee of the South Carolina Medical Association), Charleston, S. C., September 21st, 1905.

---

WE have just received from W. B. Saunders & Company, of Philadelphia, the widely-known medical publishers, an unusually attractive illustrated catalogue of their complete list of publications. It seems to us, in glancing through this catalogue, that a list of Saunders authors is a census of the leading American and foreign authorities in every branch and specialty of medical science. And new books are being added and new editions issued with a rapidity that speaks well for the success and progressiveness of the house. While comparisons are always odious, still we feel it but justice to say that in the presentation of facts about the books listed that a probable buyer wishes to know, and also for beauty and durability of mechanical get-up, this catalogue surpasses anything we have heretofore seen. It is truly representative of the house. We understand a copy will be sent free upon request.

---

CHANGES ITS NAME.—With the January issue the alkaloidal clinic changes its name to one that more fully embodies the scope of their work, namely: *The Medical Journal of Clinical Medicine.* Several departments of specialties are to be added and others will follow as arrangements can be made for them. With this additional force and a broader platform they propose to improve their excellent journal.

---

### ANNOUNCEMENT.

Messrs. Lea Brothers & Co. have pleasure in announcing for publication early in January, 1906, a completely new work on Dietetics adapted to the use of Practitioners and Students of Medicine, Nurses and the Laity.

FOOD IN HEALTH AND DISEASE. By Robert F. Williams, M.A., M.D., Professor of Principles and Practice of Medicine in the Medical College of Richmond, Virginia.

The volume will be a convenient 12mo of about 350 pages. Its price has not yet been fixed, but it will probably be about $2.00, net, delivered to any address.

It is divided for convenience, into two parts: Part I. dealing with Food in Health, and Part II. with Food in Disease.

In Part I. the needs of the body for different kinds of foods and the manner in which they are utilized are explained. The principles of cooking foods and detailed descriptions of the different articles of food in common use are given, with chapters on the proper nutriment of infants, children, adults, and the aged.

Part II. deals with the variations from the normal diet in health, necessitated by the more common diseases, and includes a chapter on general methods to be observed in feeding the sick, as well as the special directions for nourishment in diseases of different kinds.

There exists today a need for a small practical book on foods and how they should be used, which will give the facts, as known today, in a brief and clear manner, with the fewest possible technical terms.

The importance of a work of this kind, which is simple enough for a child to read and yet absolutely trustworthy and based upon the scientific achievements of accepted leading authorities, is obvious. Such books are in line with the best principles of hygiene and make for the betterment of the present as well as future generations.

